# Stone cold stroke: A case report of calcific emboli in an ischemic stroke

**DOI:** 10.1016/j.radcr.2025.01.028

**Published:** 2025-03-08

**Authors:** Anjalie Gulati, Andres Rodriguez Sein, Shibin Mathews, Levi Elhadad, Daniel Masri

**Affiliations:** a University of California, Riverside, United States; b Maimonides Medical Center, Brooklyn, NY, United States; c Tel Aviv University Faculty of Medicine, Tel Aviv-Yafo, Israel

**Keywords:** Calcified emboli, Carotid endarterectomy, Embolic stroke, Ischemic stroke, Stroke imaging, Stroke management

## Abstract

Ischemic strokes occur due to a reduction in blood flow to the brain, typically from an occluded artery. Embolic strokes are a subset where the blockage is caused by dislodged thrombus that travels from distal areas of the body, such as the heart or large arteries, and lodges in a cerebral vessel, leading to localized ischemia. While emboli are common causes of ischemic strokes, calcific emboli causing an ischemic stroke are much less reported and are frequently underdiagnosed. We report here a case of a patient with stroke symptoms who was found to have a carotid plaque on imaging, who then acutely worsened after admission, and subsequently found to have numerous new calcified emboli on a follow-up head CT.

## Introduction

Embolic strokes, a type of ischemic stroke, occur when a blood clot or material dislodges (embolizes) from a distant site, such as the heart or large arteries, and occludes a cerebral vessel, leading to cerebral ischemia. While emboli are often the cause of ischemic stroke, calcified emboli represent an under-recognized etiology. Calcific emboli are primarily associated with severe vascular calcification, such as located in a carotid or aortic plaque, and carry a heightened risk due to their rigidity and potential for recurrent embolic events. Recognizing, differentiating, and intervening with calcific emboli is crucial, as it can greatly influence management and outcomes. Advanced imaging modalities and a high index of suspicion are essential for timely detection and appropriate management, aiming to reduce the risk of recurrent strokes and improve patient outcomes.

### Case Report

We present the case of a 74-year-old female who arrived at the emergency department at 4:00pm with several hours of a right-sided facial droop and right-sided weakness. Her past medical history included hypertension, congestive heart failure, diabetes mellitus, thyroidectomy, and prior melanoma. The patient was last known to be well between 9:00 PM and 10:00 PM (19-20 h prior to presentation) the previous evening.

On arrival in the ED, neurologic evaluation on the Modified Rankin Scale (mRS) for Neurologic Disability scored the patient as an mRS 0 (no significant symptoms) and NIH Stroke Scale/Score (NIHSS) of 3 (minor stroke). A head CT performed on presentation was generally unremarkable - it showed no acute infarct, intracranial hemorrhage, or mass lesion, but revealed patchy periventricular white matter hypodensity, evidence of a chronic left basal ganglia infarct, and CNS calcific atherosclerosis. The patient was outside the window for tissue plasminogen activator (tPA) or tenecteplase (TNK), and given the suspicion for a minor ischemic stroke, she was started on dual antiplatelet therapy (DAPT) and admitted for monitoring.

The patient's family noticed worsening right sided weakness at 10:00pm (6 h following initial presentation). MRA head with and without contrast was performed at 1:30am (9.5 h after initial presentation) which showed no large vessel occlusion (LVO) or medium vessel occlusion (MeVO). MRA neck with and without contrast demonstrated a plaque resulting in mild/moderate stenosis with a questionable filling defect only seen on non-contrast time of flight (TOF) imaging, suspicious for an intraluminal thrombus. MRA brain without contrast revealed scattered foci of restricted diffusion involving the left frontal, temporal, parietal, and occipital lobes without involvement of the right cerebral hemisphere. These findings were felt to represent embolic strokes.

**Fig. 1:**
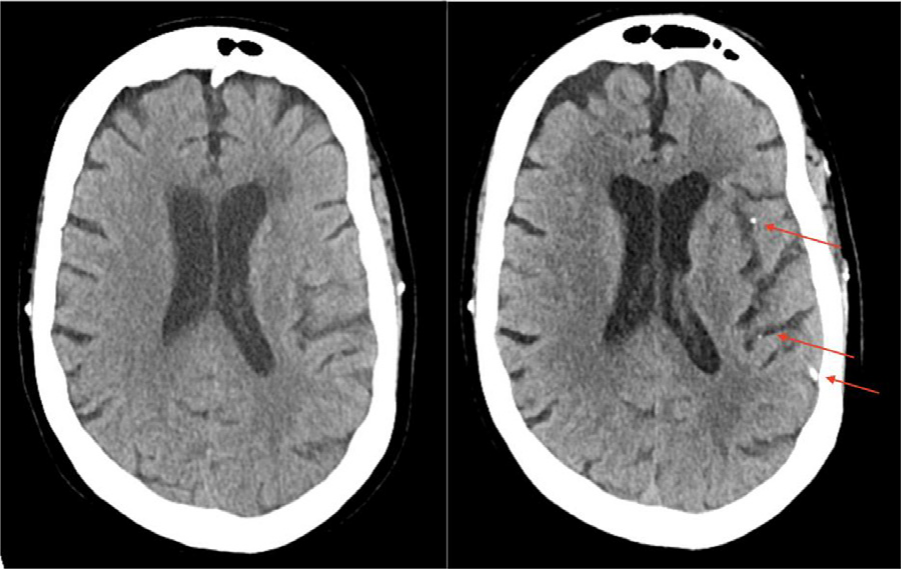
Initial unremarkable axial CT head without contrast on presentation (left); axial MIP reconstruction of follow-up CT head without contrast performed 13 h later showing numerous, new calcified emboli on a single MIP image, with additional calcifications seen on other slices (right).

The next morning at 5:00am (13 h after presentation), the patient's neurological condition worsened significantly, with new findings including slurred speech/nonverbal status, worsened weakness throughout her right-sided extremities, gait instability, a right visual field cut, and left gaze preference. At this time, her NIH Stroke Scale/Score (NIHSS) was revised to 23 (severe stroke), and she was started on heparin.

**Fig. 2:**
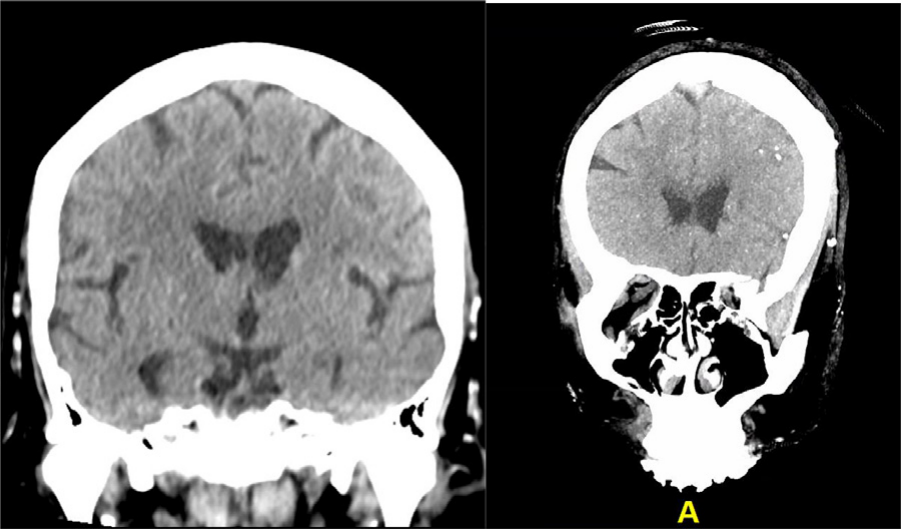
Initial unremarkable coronal CT head without contrast (left); coronal MIP reconstruction of follow up CT head without contrast performed 13 h later showing numerous, new calcified emboli on a single MIP image (right).

**Fig. 3:**
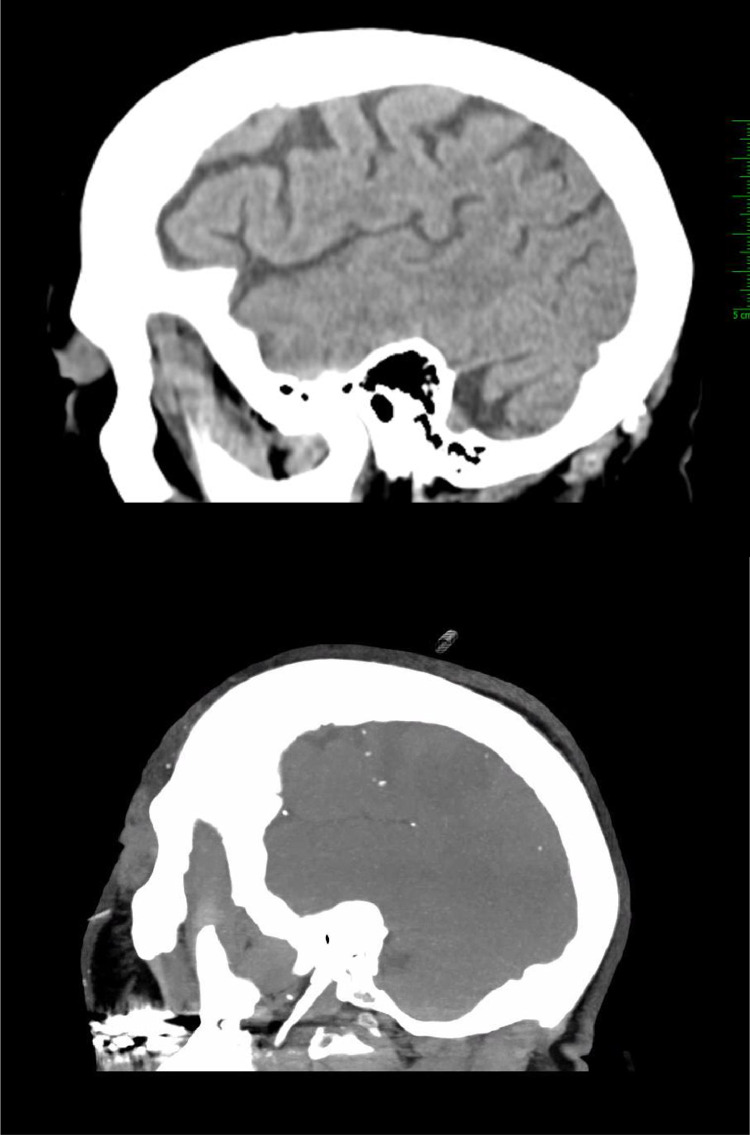
Initial unremarkable sagittal CT head without contrast (left); sagittal MIP reconstruction of follow up CT head without contrast performed 13 h later showing numerous, new calcifications on a single MIP image (right).

A repeat head CT showed new, significant findings, most notably new punctate hyperdensities with associated parenchymal hypodensities in the left cerebral hemisphere, most compatible with acute infarcts secondary to calcified emboli (Fig. 1, Fig. 2, Fig. 3).

**Fig. 4:**
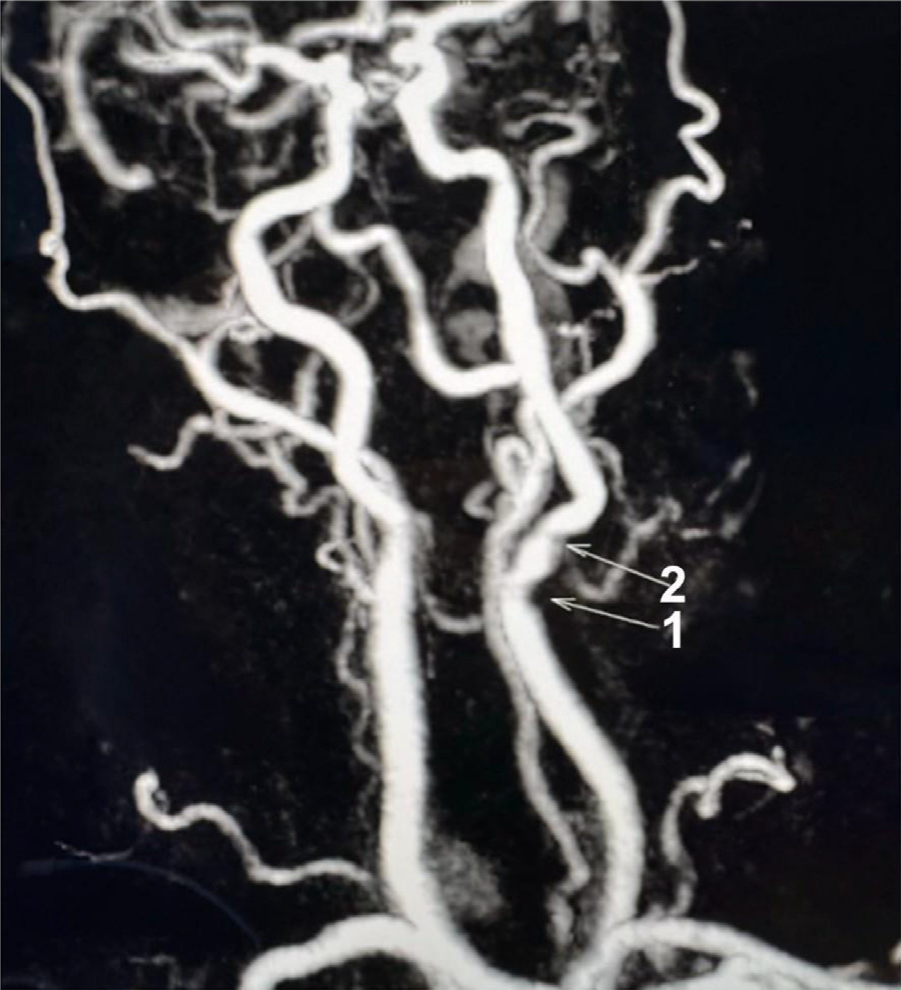
MRA Neck MIP. (1) denotes a lower calcified plaque. (2) denotes a higher calcified plaque, likely embolized.

**Fig. 5:**
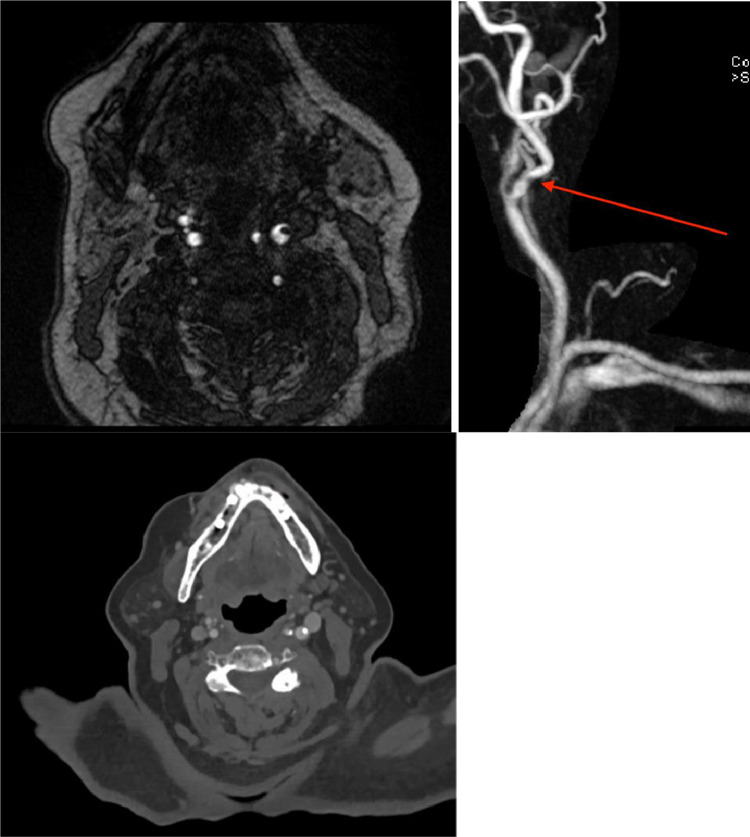
As pictured above: 9.5 h after presentation, MRA Neck demonstrating a lower filling defect in the left carotid lumen, likely present (left), but not as well-seen on the post-contrast MRA imaging of the left internal carotid artery (middle) with the CTA (right) also matching the location of the plaque.

**Fig. 6:**
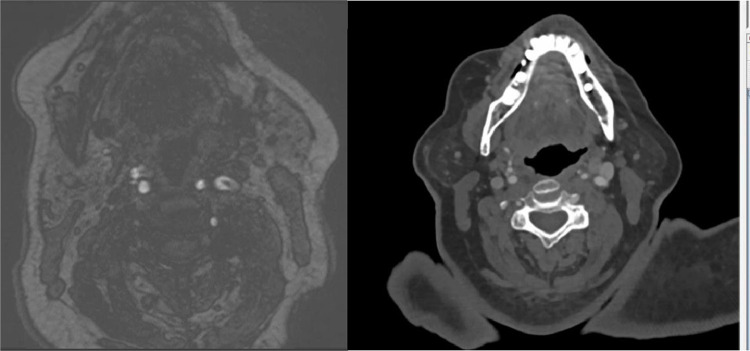
MRA neck (left) demonstrating higher plaque than seen in (1). There is no intraluminal plaque at the same level on CTA performed later, indicating this as the possible source of the dislodged plaque.

A CTA done at this time did not show the left common carotid filling defect or plaque in the lumen previously seen on MRA neck. This is presumably the source which embolized and resulted in the hyperdense calcific emboli seen on the follow up CT head (though the possibility of an alternative source of emboli cannot be excluded). The CTA demonstrated no large vessel occlusion (LVO), patent right and left common carotid arteries, as well as patent right and left internal carotid arteries with <25 % stenosis throughout. The cerebral arteries, including the bilateral ACAs, MCAs, and PCAs, were patent. Atherosclerosis of the aortic arch was also noted (Fig. 4, Fig. 5, Fig. 6).

**Fig. 7:**
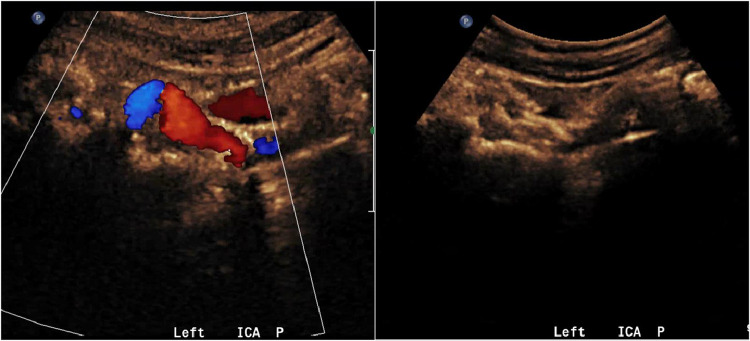
Carotid ultrasound demonstrating calcified plaque formation along the bulb with shadowing.

A transthoracic echocardiogram (TTE) done concurrently demonstrated normal left ventricular ejection fraction (66-70 %), indicating the left ventricle was functioning well and unlikely the source of the calcific emboli in this patient (Fig. 7).

### Management and Outcome

**Fig. 8:**
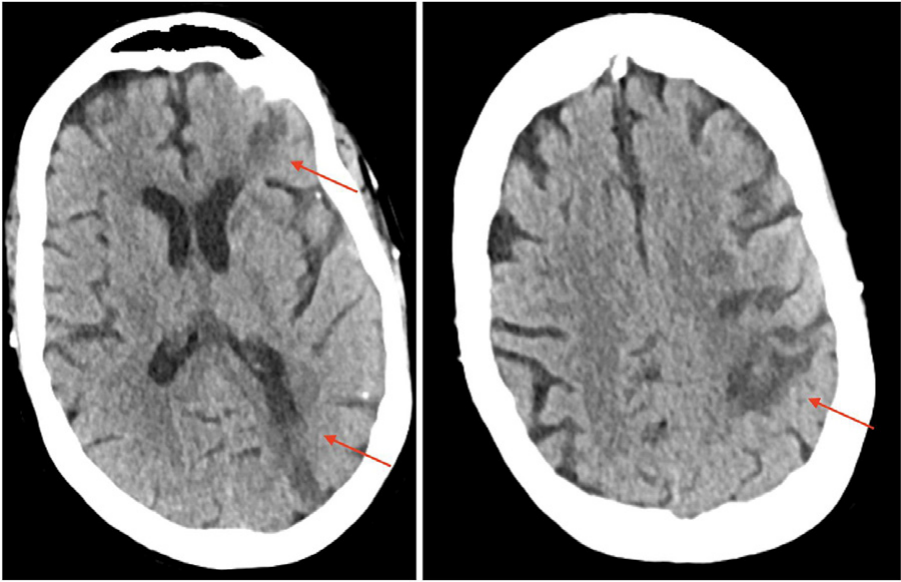
9 days post admission, axial CT heads without contrast demonstrating expected evolution of multiple left hemispheric acute/subacute infarcts. Image on left demonstrates left-sided temporo-occipital and frontal infarcts, while the right image demonstrates temporo-occipital infarct.

Initially, the patient was managed with dual antiplatelet therapy (DAPT) due to being outside the tPA/TNK window. A follow-up MRI and CT revealed punctate infarcts secondary to calcific emboli, with worsening of her neurologic exam. Eight days following admission, she underwent a left carotid endarterectomy. The operative note from the vascular surgeon described “coral-reef-like intimal plaque noted in the proximal internal carotid causing moderate stenosis. The plaque appeared friable and easily broke into pieces even on gentle touch with forceps.” This finding further supported the evidence that the patient's calcific emboli originated from the left carotid plaque. The patient was subsequently discharged for follow-up with the stroke clinic (Fig. 8).

## Discussion

In the setting of calcified cerebral emboli originating from embolization of a carotid plaque, findings could be misinterpreted as microhemorrhages - as hemorrhagic conversion may be a complication of stroke, especially if Hounsfield Units (HU) are not considered in image interpretation. While calcified cerebral emboli h8ave been considered rare in the past, recent studies have identified that calcified emboli account for 5.9% of ischemic strokes [1]. The sources of these emboli most commonly include aortic stenosis, carotid plaques (as was likely in our patient), or mitral annular calcification [2]. In this particular instance, the calcified plaque in the carotid artery likely embolized, leading to multiple punctate calcific emboli and infarcts on the ipsilateral side. Due to the appearance of the left common carotid plaque on the MRA, the calcified thrombus may have been present and visible prior to her sudden clinical decline with worsening on the NIH Stroke Scale/Score (NIHSS).

The potential to intervene earlier in this patient with carotid endarterectomy raises important questions regarding management and timing in similar cases. Calcified plaques, once dislodged, pose a significant risk for recurrent ischemic events, with studies suggesting recurrent stroke rates as high as 43% [2]. Had the calcified thrombus been recognized prior to embolization, surgical intervention such as carotid endarterectomy might have been considered to prevent further embolic events. The role of carotid endarterectomy in managing symptomatic carotid stenosis is well-established, particularly when there is evidence of a vulnerable plaque at risk for embolization. This patient was started on antithrombotic therapy, which, while effective for many thromboembolic events, was unlikely to mitigate her risk with calcified plaques.

This case demonstrated the importance of promptly identifying calcified emboli, as these patients are at a higher risk for further embolic events. Rapid identification of the embolic source, particularly if it originates from the carotid artery, is essential for intervention. Once the patient is stable, they would likely benefit from an endarterectomy to mitigate the risk of subsequent events.

## Conclusion

In conclusion, while strokes are common, calcified emboli are much rarer. Radiologists must remain vigilant for new calcifications, especially when they are identified for the first time in comparison to prior exams. It is crucial to differentiate these new calcifications from hemorrhage in the context of acute stroke, as this confusion can lead to mismanagement. Patients with calcified emboli are at a higher risk than those with non-calcified embolic disease, so early identification is vital for improving management and outcomes.

## Patient consent

Consent for publication of their case was obtained from the patient.
